# Barriers to access second-trimester abortion: A case report

**DOI:** 10.4102/safp.v63i1.5028

**Published:** 2021-01-27

**Authors:** Ramprakash Kaswa

**Affiliations:** 1 Department of Family Medicine and Rural Health, Faculty of Health Sciences, Walter Sisulu University, Mthatha, South Africa

**Keywords:** Abortion, termination of pregnancy, CTOP Act, Illegal, Second Trimester

## Abstract

Despite the implementation of the *Choice on Termination of Pregnancy* (CTOP) *Act*, many women continue to procure illegal abortions in South Africa. A lack of knowledge of the CTOP Act and poor access to legal abortion in public health facilities is a big challenge. In the scope of the CTOP Act, the termination of pregnancy is a time-restricted health service, and women presented to a health care facility in the second trimester have encounter more obstacles to access the services.

## Introduction

South Africa (SA) remains committed to achieving sexual and reproductive health freedom in an equitable and rights-based approach. South Africa’s *Choice on Termination of Pregnancy* (CTOP) *Act* of 1996 was a major step towards achieving sexual and reproductive health rights.^1,2^ Since the implementation of the CTOP Act, abortion-related deaths have decreased dramatically in South Africa. The Act serves as a global model for the reform of abortion laws.^3^ The CTOP Act had been amended in 2004 and 2008, to expand service access and to include trained nurses to work alongside midwives in providing safe abortions.^4^

The CTOP Act enables a woman of any age to access termination of pregnancy TOP services on request during the first 12 weeks of gestation. In certain cases, access is extended up to 20 weeks where pregnancy is due to rape, incest, is threatening the health of the mother and/or fetus, or socio-economic hardship. After 20 weeks of gestation legal termination of pregnancy are available in very exceptional and life-threatening circumstances with the agreement of at least two medical practitioners.^2,4^

Despite the CTOP Act, there are still significant barriers to accessing second-trimester abortion services in SA. When women are denied legal abortion in a designated facility on the grounds of the gestational age of the fetus, whether these women seek an illegal abortion or carry the pregnancy to term has never been documented.^5,6^ The decisions to continue the pregnancy or to seeking help outside the established legal health system may have serious implications on women’s reproductive health and well-being. The questions of why women seeking termination of pregnancy (TOP) outside the established legal facility in the second trimester also remains unanswered. The purpose of this case report is to highlight barriers women experience when trying to access TOP services within the scope of the CTOP Act of 1996 SA. This case study is used to illustrate the profound effect on a women’s reproductive health when a legal TOP in the second trimester is denied.

## Case

A 28-year-old G5P4A2 patient was brought to the accident and emergency (A & E) department of our hospital complaining of severe lower abdomen pain which had started earlier in the morning. She reported a recent backstreet abortion for her unwanted pregnancy. According to the patient, two weeks before the visit to A & E she visited a public health facility requesting a TOP on the grounds of socio-economic hardship. She was turned away because she was already in the second trimester of her pregnancy. She approached the backstreet abortion provider and took some abortifacients tablets for termination of this unwanted pregnancy. The next day she started bleeding heavy per vagina, so she approached the same place. They reassured her that everything was fine and send back home. On the third day after the illegal termination, she continued to bleed and develop severe lower abdomen pain and weakness. When a family member saw the patient had almost collapsed at home due to severe pain, they brought her to the hospital.

On presentation to our hospital, she was conscious, pale, and in obvious severe pain, her blood pressure (BP) was 105/74 millimeter of mercury (mmHg), pulse 106/min, the temperature was 37.3 °C. An abdominal examination revealed lower abdomen tenderness. Bowel sounds were normal. On per vagina examination, there was active bleeding, the uterus was about 16 weeks’ size, mobile and cervix was open 4–5 cm. Her blood investigation showed Hb 4 mmol/dl, white cell count 12.5 × 109/l, platelets were 287 × 109/l. Urea, creatinine, and electrolytes were all within normal limits. Ultrasound pelvis showed a singleton intrauterine pregnancy with a gestational age of 17 weeks 3 days and no fetal cardiac activity. A clinical diagnosis of septic abortion and severe anemia due to acute blood loss was made. An emergency surgical evacuation was planned after receiving three units of packed red cell transfusion. Post-procedure she received broad-spectrum antibiotics and intravenous fluids. She responded well to treatment and on the third day, she was discharged on an oral antibiotic. She attended counseling and family planning during a follow-up visit at a women’s clinic.

## Discussion

About 20% to 25% of TOPs in South Africa (SA) are performed during the second trimester.^7^ According to the Amnesty International 2017 report, only 264 out of the 3880 public health facilities in SA provide second-trimester abortion services. The report also highlighted the fact that there were no public facilities in the Eastern Cape that offered second-trimester abortion services. Many women who requested second-trimester abortions get turned away at the public health facilities. A study conducted in Cape Town during 2015 reported that 20% of women requesting TOP in their second trimester of pregnancy were denied this service.^8^

Despite the liberalization of the abortion law and the relative availability of abortion facilities in many parts of urban SA, access to second-trimester legal abortion services remains a challenge for the vast majority of rural South African women.^9,10,11^ As a result of this, there is an unacceptably high rate of illegal abortion among adolescent girls, especially in rural areas. This high number of illegal abortions indicates that legalization alone cannot ensure sexual and reproductive health freedom.^6^

Only a few studies in SA have been reported the complex reason why women seek TOPs outside of designated health facilities in the second trimester. Factors include lack of knowledge of the CTOP Act, shortage of public health service providers, and refusal by health care service providers.^9^ A lack of knowledge on abortion rights under the CTOP Act and the perceived poor quality of reproductive health services in the designated facilities are the most important barriers to access.^8^ The lack of knowledge of the time-limited nature of the TOP services forced the disgruntled women seeking help outside the established legal system.^6,10,12^ According to the study reported by Harris and Grossmann, women who seeking TOP services outside the designated facility had poor health outcomes.^5^

A second-trimester abortion is a time-restricted procedure and is particularly challenging due to the health expertise required. The most frequent reason for women seeking TOP services in the second trimester is based on the ground of socio-economic hardship. The CTOP Act leaves considerable scope for interpretation by health professionals.^4,5^

In response to these challenges, there is an urgent need to implement the CTOP Act in a standardized and expanded manner, thus reaffirming reproductive health freedom as per the Constitution. This may influence the overall ability of the CTOP Act to reduce abortion-related morbidity and mortality. It marks 21 years since our landmark legislation of the CTOP Act was enacted, and women are still demanding the rights that the Act provides for them. It is a legislative demand that abortion services in the scope of the CTOP Act should be accessible and available to all women in South Africa.

## Conclusion

The availability of second-trimester abortion services in public health facilities in the Eastern Cape is limited. When termination of pregnancy is denied at legal facilities, women turn to seek options beyond the legal abortion services. There is an urgent need to address barriers of access to legal abortion services in the scope of the CTOP Act in public health facilities, especially in second-trimester abortion.
